# Susceptibility of Selected Tea Shoots to Oviposition by *Empoasca onukii* (Hemiptera: Cicadellidae) and Feasibility of Egg Removal with Harvesting

**DOI:** 10.3390/insects11060338

**Published:** 2020-06-01

**Authors:** Bo-Hua Hou, Hao Tang, Jian-Long Li, Xiang Meng, Ge-Cheng Ouyang

**Affiliations:** 1 Guangdong Key Laboratory of Animal Conservation and Resource Utilization, Guangdong Public Laboratory of Wild Animal Conservation and Utilization, Guangdong Institute of Applied Biological Resources, Guangzhou 510260, China; mengxiangxs@126.com; 2 Tea Research Institute, Guangdong Academy of Agricultural Sciences, Guangzhou 510640, China; teaplanting@163.com (H.T.); skylong.41@163.com (J.-L.L.)

**Keywords:** oviposition preference, leaf order interval, pest incidence, leafhopper, shoot characteristics

## Abstract

The *Empoasca onukii* (Hemiptera: Cicadellidae) female lays its eggs inside the epidermis of the tea plant shoots. This has led to speculation that shoot harvesting could represent a method of egg removal. To verify the validity of this hypothesis, we sought to determine which part of the shoot was used for the oviposition and how the value of the harvested shoot affects the cost of the egg removal. In this study, four tea cultivars were chosen to examine the preferences for the site of oviposition. In addition, a mathematical model was used to describe the correlation between the economic value of the selected shoot and eggs laid within the shoot. Our study revealed that the pest preferred the 3rd and 4th leaf order intervals of the shoot as the oviposition sites, and the oviposition preferences was dependent on the leaf order interval class across all tea cultivars. In addition, a significant negative exponential relationship was found between the economic value of the selected shoot and the percentage of the eggs laid within the shoot, indicating that egg removal through shoot harvesting was limited. The findings of this study could be used to better understand the role of shoot harvesting in egg removal and would provide new insights into the understanding of the incidence of this pest.

## 1. Introduction

The tea green leafhopper *Empoasca* (*Matsumurasca*) *onukii* Matsuda (Hemiptera: Cicadellidae), formerly known as *Empoasca flavescens* Fabricius, and *Empoasca vitis* (Göthe), is one of the most destructive pests in the tea plant *Camellia sinensis* (L.) Kuntze (Theaceae) in East Asia [1,2,3,4], where tender shoots of the tea plant are periodically harvested [5]. *E. onukii* lays its eggs inside the epidermis of the shoots [6,7]. The number of generations of this pest per year ranges from 10 to 17, depending on site-specific climatic conditions, especially temperature [8]. Nymphs and adults of *E. onukii* use their stylets to pierce and suck the sap from tea buds, leaves, and shoots, which causes serious economic problems for the tea industry [9,10].

Organosynthetic pesticides have been frequently used to control the leafhopper all year round. However, these applications represent increased costs for tea growers and can have adverse effects on the environment and on natural enemy populations which can result in pest resurgence [1,11]. Alternatively, numerous other control agents and methods against this pest have been proposed, including biological control [12], physical control [13], and chemical intervention [14,15,16]. However, the management of this pest remains challenging.

As stated above, eggs of *E. onukii* are laid within the stalk and leaf petiole epidermis of the newly-growing tea plant shoots. We asked the question as to whether hand plucking, the most common harvest method of the shoot harvesting, could represent an effective method of egg removal. To answer this question, we took into account the facts that instead of the whole shoot, certain leaf order intervals of the shoot should be selected for the harvesting. In addition, generally, the more attached leaves are selected, the lower the value of the shoot, because of the reduced levels of aroma and flavor precursors [17].

In order to accurately evaluate the role of shoot harvesting in egg removal, while taking into account the actual harvesting practice and the harvested shoot value, it is necessary to determine the egg distribution within each of the leaf order intervals of the shoot, and to consider the economic value of the selected shoot on the cost of egg removal. The purpose of this study was to determine the oviposition preference of *E. onukii* in the tea plant shoot, and to illustrate the correlation between the economic value of the selected shoot and the proportion of eggs laid within the shoot.

## 2. Materials and Methods

### 2.1. Study Site

The experiment was conducted in August 2018 in the experimental tea plantation of the Tea Research Institute, Guangdong Academy of Agricultural Sciences. The tea plantation was located in north-central Guangdong, China (24.18° N, 113.23° E). This area belongs to a typical tea-growing region known as the South China Tea Region. About 150 tea cultivars were bred in this plantation. Our study took into consideration four of these cultivars: Jinxuan, Jinguanyin, Yinghong-9, and Yunnandaye, all of which were widely planted in this area. Each cultivar was cultivated in rows of eight-year-old plants in an approximately 0.2 hectare field. Plants were maintained as 70 cm high flat-topped foliage canopy under similar agricultural management practices.

### 2.2. Measurement of Cultivar Characteristics

**Figure 1:**
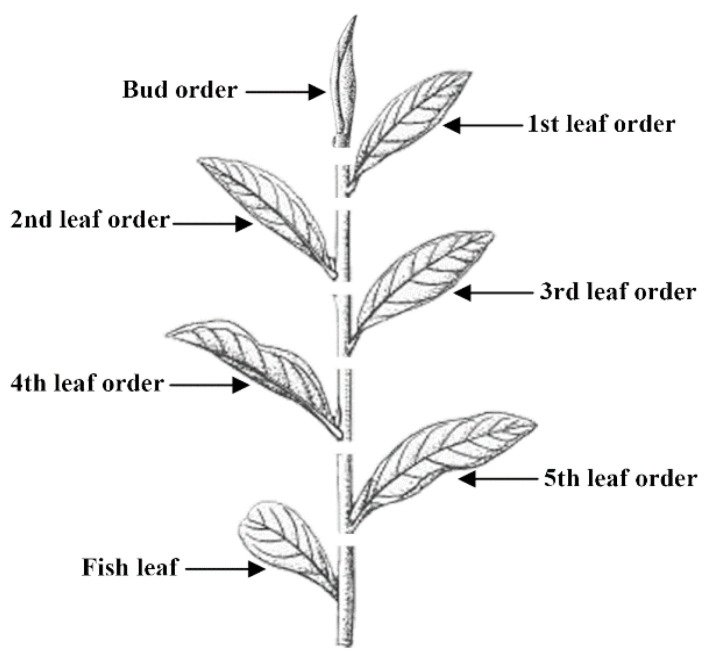
A typical newly growing shoot of the tea plant. The whole shoot was classed as the corresponding leaf order intervals according to the shoot harvesting method.

The newly-growing shoot with a bud and more than four attached leaves was chosen for the measurement. According to the method of shoot harvesting, certain leaf order intervals of the shoot should be selected for harvesting. Here, we use the word “leaf order interval” to describe the bud or the attached leaf and its stem that is selected for harvesting. The whole shoot therefore was classed as the bud order interval, the 1st leaf order interval, the 2nd leaf order interval, etc. (Figure 1). The length of each of the leaf order intervals ranged from the bud order interval to the 4th leaf order interval and was measured as a principal characteristic to differentiate these four cultivars. Thirty shoots (3 rows with 10 shoots per row) per cultivar were randomly sampled for the experiment.

### 2.3. Examination of Eggs

**Figure 2:**
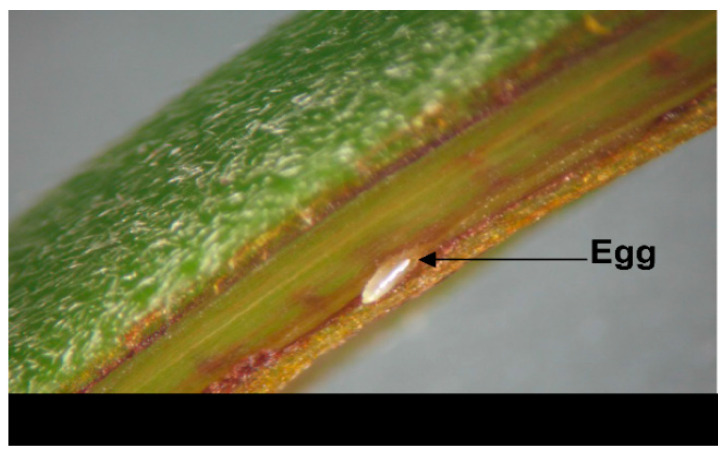
An egg of *Empoasca onukii* within the epidermis of the tea plant shoot.

Ninety shoots (3 rows with 30 shoots per row) for each cultivar were randomly sampled from the field to examine the distribution of the eggs laid by the leafhopper. Eggs were examined by dissecting the stalk and leaf petiole epidermis of the shoot under a dissection microscope (Figure 2). The number of eggs present within each leaf order interval class was recorded. Samples were collected and egg examination was completed within 48 hours, at the same time for each cultivar.

### 2.4. Collection and Analysis of Economic Data

The economic data on the selected shoots was collected from the experimental tea plantation of the Tea Research Institute, Guangdong Academy of Agricultural Sciences. In this study, the economic value of the selected shoot was expressed as value per unit weight. The relationship between the economic value of the selected shoot and the percentage of the eggs within the shoot was described through curve estimation.

### 2.5. Preparation of Data and Statistical Analyses

The length of each of the leaf order interval classes was measured and analyzed by one-way ANOVA and post hoc Tukey’s test to compare the differences among the four cultivars in the same leaf order interval class at the *α* = 0.05 level. The number of eggs within each leaf order interval was converted into a percentage, and the percentage values were normalized by arcsine square root transformation prior to analysis, then, an analysis similar to that adopted for the interval length was performed to test the variation of the eggs among the leaf order interval classes in each cultivar. A two-way ANOVA with leaf order interval and cultivar as factors was also used to determine the variation in the distribution of eggs. Curve estimation was used to extract the best fit correlation between the economic value of the selected shoot and the percentage of the eggs within the shoot. All statistical analyses were performed using IBM SPSS Statistics version 22.0 software (IBM Corp., Armonk, NY, USA).

## 3. Results

### 3.1. Cultivar Characteristics

**Figure 3:**
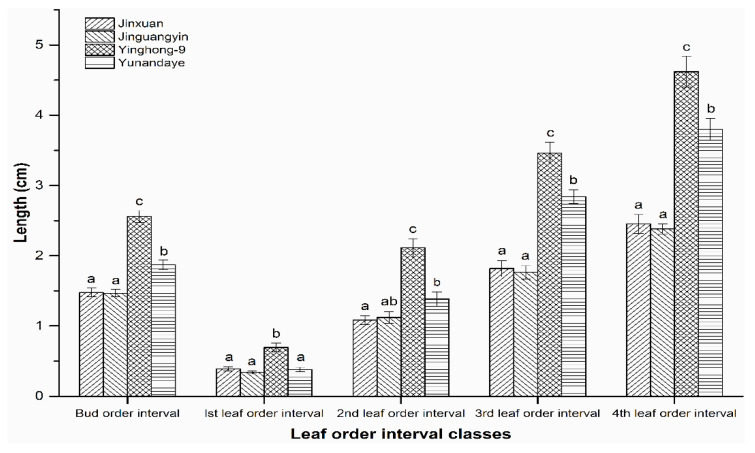
Length of the different leaf order intervals of the tea plant shoot. Bars represent mean ± SE of 30 samples for each cultivar. Bars with the same letters indicate that means comparisons were not significantly different in the same leaf order interval class of the four cultivars (ANOVA, Tukey’s test, *p* > 0.05). (Original data: Table S1.)

The leaf order interval length differed significantly among these four cultivars in the leaf order interval classes (bud order interval: *F* = 56.98; df = 3, 116; *p* < 0.001; the 1st leaf order interval: *F* = 16.19; df = 3, 116; *p* < 0.001, the 2nd leaf order interval: *F* = 24.08; df = 3, 116; *p* < 0.001, the 3rd leaf order interval: *F* = 50.13; df = 3, 116; *p* < 0.001, and the 4th leaf order interval: *F* = 49.06; df = 3, 116; *p* < 0.001). Cultivars Jinxuan and Jinguanyin had the shortest leaf order interval length, Yinghong-9 had the longest leaf order interval length, and Yunnandaye had an intermediate leaf order interval length. No significant difference was observed among Yunnandaye, Jinxuan, and Jinguanyin in the 1st leaf order interval length, additionally, similar length was observed between Yunnandaye and Jinguanyin in the 2nd leaf order interval (Figure 3 and Table S1).

### 3.2. Egg Distribution

**Figure 4:**
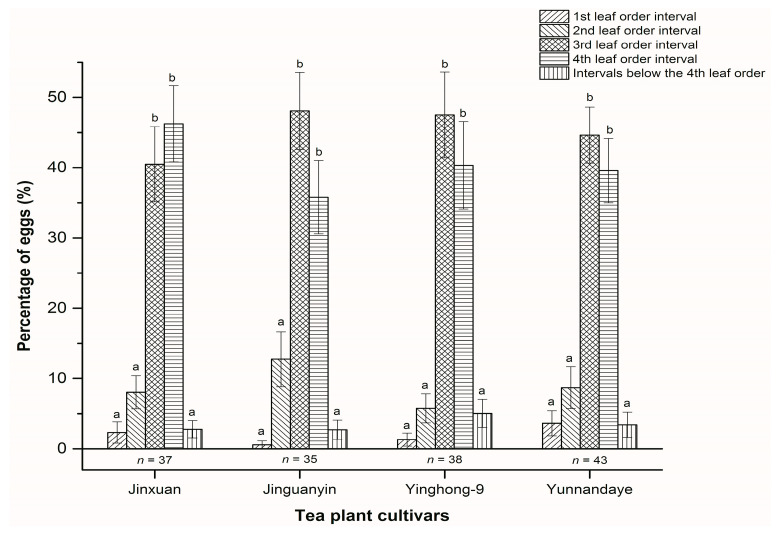
Percentage of *Empoasca onukii* eggs in the different leaf order intervals of the tea plant shoot. Bars represented mean ± SE of the infested samples (*n* = 37, 35, 38, and 43 shoots sampled with a total of 172, 128, 111, and 171 eggs for Jinxuan, Jinguanyin, Yinghong-9, and Yunnandaye cultivars, respectively). Bars with the same letters indicate that means comparisons were not significantly different among the leaf order interval classes in a cultivar (ANOVA, Tukey’s test, *p* > 0.05). (Original data: Table S2.)

In total, 37, 35, 38, and 43 of 90 sampled shoots were infested with a total of 172, 128, 111, and 171 eggs of the leafhopper for the Jinxuan, Jinguanyin, Yinghong-9 and Yunnandaye cultivars, respectively. No eggs were found within the bud order interval for any cultivar. For the remainder of the leaf order interval classes, the percentage of eggs differed significantly in the four cultivars (Jinxuan: *F* = 35.58; df = 4, 180; *p* < 0.001, Jinguanyin: *F* = 29.45; df = 4, 170; *p* < 0.001, Yinghong-9: *F* = 28.64; df = 4, 185; *p* < 0.001, and Yunnandaye: *F* = 40.00; df = 4, 210; *p* < 0.001). For each cultivar, the lower percentages of eggs was detected within the 1st and 2nd leaf order interval classes, as well as within the leaf order interval classes below the 4th one. Most of the eggs were found within the 3rd and 4th leaf order interval classes (Figure 4 and Table S2). Two-way ANOVA analyses indicated that there was no statistically-significant interaction between leaf order interval and tea cultivar in the distribution of eggs (*F* = 0.73; df = 12, 745; *p* = 0.723). Tea cultivar had no significant effect on the distribution of eggs (*F* = 0.00; df = 3, 745; *p* = 1.00), whereas the distribution of eggs depended on leaf order interval class (*F* = 128.46; df = 4, 745; *p* < 0.001).

### 3.3. Economic Value and Egg Removal

The economic value of the selected shoot is shown in Table 1. Shoots with more attached leaves had a lower economic value. For the Jingxuan cultivar, when the shoot was selected as the bud only, the economic value of the shoot was 24.00 Chinese Yuan (CNY) per kg. Accordingly, the percentage of the eggs within this shoot was zero, indicating that no eggs would be removed through harvesting. Again, when the shoot was selected as one leaf attached, the economic value of the shoot was 14.00 CNY per kg. Accordingly, the cumulative percentage of eggs within this shoot was 2.32% (0 in the bud order interval, plus 2.32% in the 1st leaf order interval), indicating that 2.32% of the eggs would be removed through harvesting, and so on (Table 1 and Figure 4).

**Figure 5:**
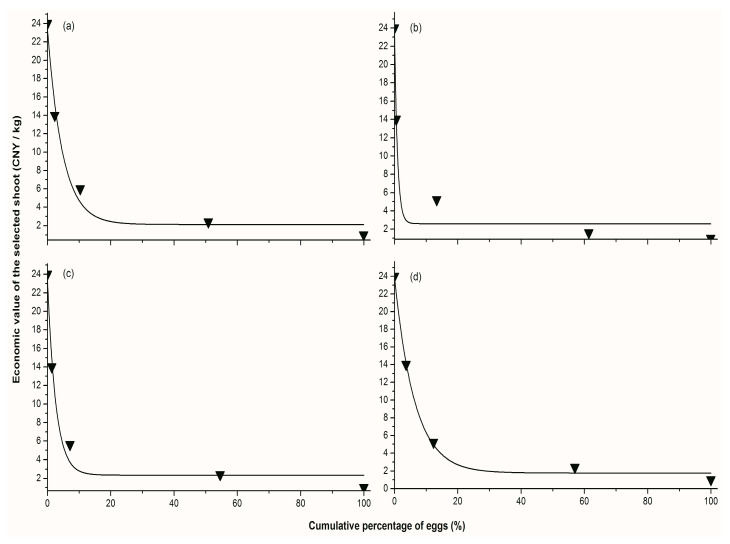
The relationship between the economic value of the selected shoot and the cumulative percentage of eggs of *Empoasca onukii* within the shoot. The figure shows the exponential model fitting the data for each cultivar: (**a**) Jinxuan, (**b**) Jinguanyin, (**c**) Yinghong-9, and (**d**) Yunnandaye.

For each cultivar, curve estimation was used to extract the best fit correlation between the economic value of the selected shoot and the percentage of the eggs within the shoot. The mathematical models, such as linear, logarithmic, inverse, quadratic, cubic, power, compound, S-curve, growth, and exponential models in SPSS were fitted to the data. Overall, the exponential model showed the best regression fit and performed better than the other models (Figure 5 and Table 2).

## 4. Discussion

As eggs of *E. onukii* are laid within the tea plant shoot, and certain leaf order intervals of the shoot are selected for the harvesting, the leaf order interval length was considered as a determinant affecting the egg distribution in this study. The results of this study demonstrated that the length differed significantly among the four cultivars in the same leaf order interval class, however, the proportion of eggs in the same leaf order interval class was similar among cultivars. The findings indicated that the distribution of eggs was dependent on the leaf order interval class regardless of cultivar. 

Little was known about the modalities of *E. onukii* oviposition behavior [18,19]. The results of this study indicated that most of the eggs were laid within the 3rd and 4th leaf order interval classes, whereas a low proportion of the eggs were detected within the leaf order interval classes below the 4th leaf order interval. Presumably, shoot toughness might be an important determinant of egg-laying behavior. This could be explained by the previous observation that there was a sharp decrease of shoot tenderness from the upper internodes to the lower internodes in tea plants [20].

Interestingly, the 1st and 2nd leaf order interval classes probably had higher tenderness than those of the 3rd and 4th leaf order interval classes [20]. However, the results in this study revealed that these two interval classes were less-frequently used for oviposition, which could be due to the thickness of the epidermis. Here, we did not consider the relationship between the epidermal thickness and the oviposition preference. In the vineyard, eggs of *E. vitis* were reportedly laid in the 1st or 2nd order vein of the grape leaf, as the major veins were thicker than small veins [21]. Another study found that the thicker cotton leaf veins were observed to increase the oviposition response of the cotton leafhopper *Amrasca biguttula* (Ishida) (Hemiptera: Cicadellidae) [22]. Both of the previous findings support the idea that thicker veins might stimulate increased egg-laying in leafhoppers. Eggs of *E. onukii* are laid within the shoot epidermis instead of leaf veins in tea plants. The epidermal thickness in the uppermost part is thinner than that in the lower parts of the tea plant shoot [23], which led to the result that the bud order, 1st leaf order, and 2nd leaf order interval classes were less frequently used for oviposition.

We recognized that the distribution of eggs within the leaf order interval was a strong predictor of the oviposition preference of *E. onukii*. These findings could prove useful for both the monitoring methods and the controlling techniques for this pest. For example, egg density of the 3rd and 4th leaf order intervals could be used to indicate pest infestation level, and to predict the abundance of subsequent generations. Moreover, it is likely that the pest would be suppressed as the oviposition sites were destroyed. The data on the distribution of eggs was drawn from one season point, the abundance of the leafhopper in the field was unknown in this study. Would seasonal variation exert an effect on the egg distribution? Further evidence is needed to confirm these findings.

Specimens collected in Chinese tea plantations indicated that no leafhopper species other than *E. onukii* are pests of tea plants [2,3]. Several hypotheses have attempted to explain the incidence of this pest. These hypotheses include: (1) high fecundity of this species [5], (2) widely-available host plants [18], (3) insecticide resistance [1,11], and (4) few effective natural enemies [14]. These theoretical and experimental studies were essential for understanding the incidence of this pest. However, we should not neglect the economic factor in our quest to understand this issue. The constructed mathematical model demonstrates that egg removal through shoot harvesting was likely to be of limited effectiveness considering the value of the selected shoots. These findings likely could provide new insights into understanding the incidence of this pest.

## 5. Conclusions

It is clear that eggs of *E. onukii* are laid within the newly-growing tea plant shoot, suggesting that the laid eggs would likely be removed through shoot harvesting. However, there is no convincing evidence that shoot harvesting could represent an effective method of egg removal. Accordingly, in the current work, we determined the oviposition preference of *E. onukii* in the tea shoot and addressed the influence of the selected shoot value on the egg removal. Based on our observation, the 3rd and 4th leaf order intervals of the shoot were most suitable for the oviposition, and the oviposition preferences were dependent on the leaf order interval class regardless of tea cultivar. In addition, the harvested shoot value significantly affected the cost of egg removal, indicating that the effectiveness of egg removal through shoot harvesting was limited. This work provides the basis for understanding the role of shoot harvesting in egg removal and may provide new insights into the understanding of the incidence of this pest.

## Figures and Tables

**Table 1:** The economic value of the tea plant shoot with certain attached leaves (CNY/kg) ^a^.

Cultivars	Bud Only	One Leaf	Two Leaves	Three Leaves	Four or More Leaves
Jinxuan	24.00	14.00	6.00	2.40	1.00
Jinguanyin	24.00	14.00	5.20	1.60	1.00
Yinghong-9	24.00	14.00	5.60	2.40	1.00
Yunnandaye	24.00	14.00	5.20	2.40	1.00

^a^ Data were collected from the experimental tea plantation of the Tea Research Institute, Guangdong Academy of Agricultural Sciences.

**Table 2:** The exponential model summary and parameter estimates.

Cultivars	Equation	F-Test of the Model					
		R	R^2^	F	df1	df2	P
Jinxuan	*y* = 13.795*e**(−0.028*x*)	0.943	0.889	23.925	1	3	0.016
Jinguanyin	*y* = 13.496*e**(−0.029*x*)	0.940	0.883	22.622	1	3	0.018
Yinghong-9	*y* = 13.114*e**(−0.027*x*)	0.932	0.868	19.740	1	3	0.021
Yunnandaye	*y* =14.012*e**(−0.028*x*)	0.940	0.884	22.814	1	3	0.017

Where, *y* is the economic value of the selected shoot, and *x* is the percentage of the eggs within the shoot.

## Supplementary Materials

The following are available online at https://www.mdpi.com/2075-4450/11/6/338/s1, Table S1: The length of the leaf order intervals, Table S2: The number of laid eggs of *Empoasca onukii* within each of the leaf order interval classes.

## References

[1] Saha D., Mukhopadhyay A. (2012). Insecticide resistance mechanisms in three sucking insect pests of tea with reference to North-East India: An appraisal. Int. J. Trop. Insect Sci..

[2] Fu J.-Y., Han B.-Y., Xiao Q. (2014). Mitochondrial COI and 16sRNA evidence for a single species hypothesis of *E. vitis*, *J. formosana* and *E. onukii* in East Asia. PLoS ONE.

[3] Qin D., Zhang L., Xiao Q., Dietrich C.H., Matsumura M. (2015). Clarification of the identity of the tea green leafhopper based on morphological comparison between Chinese and Japanese specimens. PLoS ONE.

[4] Zhang L., Wang F., Qiao L., Dietrich C.H., Matsumura M., Qin D.-Z. (2019). Population structure and genetic differentiation of tea green leafhopper, *Empoasca* (*Matsumurasca*) onukii, in China based on microsatellite markers. Sci. Rep..

[5] De Costa J., Mohotti A.J., Wijeratne M.A. (2007). Ecophysiology of tea. Braz. J. Plant Physiol..

[6] Zhu J.G., Kuang R.P., Hu H.M., Tao T., Zhu Q.Z. (1993). The development, reproduction and spatial distribution of lesser green leafhopper (*Empoasca flavescens*) on different tea cultivars. Zool. Res..

[7] Kosugi Y. (1998). Oviposition sites of tea green leafhopper, *Empoasca onukii* Matsuda in a new shoot of tea plants. Annu. Rep. Kansai Plant Prot. Soc..

[8] Zhang W.Y., Lin M.X., Zhang H.G. (1997). Relationship between temperature and development of *Empoasca vitis* Göthe (Homoptera: Cicadelidae). J. Anhui Agric. Univ..

[9] Kosugi Y. (2000). Influence of injury by tea green leafhopper, *Empoasca onukii* Matsuda on leaves in new shoots of tea plants. Chagyo Kenkyu Hokoku Tea Res. J..

[10] Yorozuya H., Tanaka J.-I. (2012). The degrees of feeding damage and the numbers of probing punctures by tea green leafhopper, *Empoasca onukii* Matsuda in tea germplasms. Kyushu Plant Prot. Res..

[11] Wei Q., Yu H.-Y., Niu C.-D., Yao R., Wu S.-F., Chen Z., Gao C.-F. (2015). Comparison of insecticide susceptibilities of *Empoasca vitis* (Hemiptera: Cicadellidae) from three main tea-growing regions in China. J. Econ. Entomol..

[12] Chen L., Yuan P., Pozsgai G., Chen P., Zhu H., You M. (2019). The impact of cover crops on the predatory mite *Anystis baccarum* (Acari, Anystidae) and the leafhopper pest *Empoasca onukii* (Hemiptera, Cicadellidae) in a tea plantation. Pest Manag. Sci..

[13] Bian L., Sun X., Luo Z., Zhang Z., Chen Z. (2014). Design and selection of trap color for capture of the tea leafhopper, *Empoasca vitis*, by orthogonal optimization. Entomol. Exp. Appl..

[14] Cai X.-M., Xu X.-X., Bian L., Luo Z.-X., Xin Z., Chen Z. (2015). Attractiveness of host volatiles combined with background visual cues to the tea leafhopper, *Empoasca vitis*. Entomol. Exp. Appl..

[15] Xu X., Cai X., Bian L., Luo Z., Li Z., Chen Z. (2017). Does background odor in tea gardens mask attractants? Screening and application of attractants for *Empoasca onukii* Matsuda. J. Econ. Entomol..

[16] Bian L., Cai X.-M., Luo Z.-X., Li Z.-Q., Xin Z.-J., Chen Z. (2018). Design of an attractant for *Empoasca onukii* (Hemiptera: Cicadellidae) based on the volatile components of fresh tea leaves. J. Econ. Entomol..

[17] Obanda M., Owuor P.O., Taylor S.J. (1997). Flavanol composition and caffeine content of green leaf as quality potential indicators of Kenyan black teas. J. Sci. Food Agric..

[18] Xin Z.-J., Li X.-W., Bian L., Sun X.-L. (2016). Tea green leafhopper, *Empoasca vitis*, chooses suitable host plants by detecting the emission level of (3Z)-hexenyl acetate. Bull. Entomol. Res..

[19] Zhang X., Pengsakul T., Tukayo M., Yu L., Fang W., Luo D. (2017). Host-location behavior of the tea green leafhopper *Empoasca vitis* Göthe (Hemiptera: Cicadellidae): Olfactory and visual effects on their orientation. Bull. Entomol. Res..

[20] Huang Y., Wei K., Wang L.Y., Cheng H., He W., Zhou J. (2012). Study of developmental pattern of tea shoot tenderness on the base of texture analyser. J. Tea Sci..

[21] Herrmann J.V., Böll S. (2001). Validation of a new method for monitoring eggs of the grape leafhopper (*Empoasca vitis*) in grapevine leaves. IOBC-WPRS Bull..

[22] Sharma A., Singh R. (2002). Oviposition preference of cotton leafhopper in relation to leaf-vein morphology. J. Appl. Entomol..

[23] Huang Y.H. (1991). Anatomy of stage developmental heterogeneity on stem of tea plant. J. Tea Sci..

